# Dis-eases of Korean nurses: a women’s health perspective

**DOI:** 10.4069/kjwhn.2021.12.07

**Published:** 2021-12-15

**Authors:** Moon Jeong Kim

**Affiliations:** Department of Nursing, Pukyong National University, Busan, Korea

As families with happy mothers are happy, patients cared for by happy nurses will have higher hospitalization satisfaction. However, just as we neglect the health of mothers who are primary caregivers at home, we tend to neglect nurses’ wellbeing in hospitals. Women’s health refers to the overall experience of women and their dis-ease, a term that was proposed with a feminist lens as a direct contrast to ‘disease’ [1]. As most nurses are women, women’s health nurses need to pay particular attention to the dis-ease experienced by nurses. This editorial aims to illustrate the dis-ease, i.e., various sources of uncomfortableness experienced by nurses nurses in South Korea (hereafter Korea), its related factors, and suggest directions for improvement. Understanding nurses’ dis-ease will be the first step to improve the quality of life of nurses and may also help to encourage nurse retention and a stable supply of nursing personnel.

## Dis-ease of nurses

According to the Korean Nurses’ Health Study (KNHS), an ongoing cohort study [2], Korean nurses’ most common physical health problem was gastrointestinal problems, with a morbidity rate of 20%. Being underweight was the next most common health problem, accounting for 15%. These problems seem to be related to occupational factors and diet [2].

Stressors that hinder nursing are long working hours and night shifts, which cause emotional and physical exhaustion, affecting the quality of life. The stresses and professional dissatisfaction inherent in nurses’ daily life are related to human complexity, work relations, responsibility, professional autonomy, and professional skills over time and abilities. Nurses’ sense of autonomy and responsibility can shift to work overload and pressure, negatively affecting their quality of life. The urgency of nursing work has a more negative impact on workers’ health. In situations of conflict and dissatisfaction with the work environment, burnout may occur due to occupational stress at the physical and psychological levels. Attributes of burnout are emotional exhaustion, depersonalization, and lack of professional achievement. Nurses consume alcohol for the purpose of relieving stress, and it was reported that 58.2% of Korean nurses regularly drink alcohol [2,3].

More than one case of suicide by nurses in Korea has been reported every year since 2010 [4]. The high suicide rate among nurses is a global phenomenon, with around 44 nurses committing suicide each year in the United Kingdom and Wales between 2011 and 2017 [5]. Considering that Korea has the highest suicide rate among Organisation for Economic Co-operation and Development (OECD) countries, there may be more cases. In Korea, even official statistical data on the suicide rate of nurses have not been made [3], and there has been no public discussion about it.

## Related factors

### The shortage of nursing personnel

While nurse shortage is a global problem, it is a severe issue in Korea, where the number of active nurses per 1,000 population is 3.8, nearly half of the OECD average of 7.4 in 2018. Shortage of practicing nurses is severe, despite the number of new nurses graduating each year is steady at 35.0 per 100,000 people, higher than the OECD average of 26.5 [6]. Such statistical data reflect the reality that despite no problems with the supply of nurses, that employment is not well maintained. According to working time regulations applied in the United Kingdom in 1998, nurses can take a break of at least 20 minutes if they work more than 6 hours. However, in reality, they could not drink a sip of water or take a break while working because of the shortage of nurses [3]. According to the Nursing and Midwifery Council in the United Kingdom, 44% of nurses retired due to working conditions in 2017 [3], which aggravated nurse shortage. The shortage of nurses are related to burnout, job dissatisfaction, and turnover intention. Reducing the nurse-patient ratio is also an urgent issue in improving nurses' quality of life and the quality of care provided by nurses.

### Shift work

Korean nurses frequently work three-time shifts on a rotating basis. As a result, 88.4% of nurses in the KNHS answered that they worked night shifts for an average of 5.3 years of their lifetime [2]. Three-time shifts, especially the night shift, was blamed for many health problems such as cardiovascular dis-eases, cancer, metabolic syndrome, diabetes, and overweight. Night shift, in particular, has been noted as a causal factor contributing to breast cancer and other cancers [7]. To relieve work-related stress, nurses need to engage in various hobbies or leisure activities. However, shift work makes it difficult [3]. Considering that other countries such as the United States, have a system where nurse can work on a fixed shift system, applying various shift models and flexible work hours needs to be applied in Korea. Furthermore, increasing the opportunities for nurses to choose work shifts according to their lifestyles would be helpful.

### Bullying

Recently, the media’s report on a nurse’s suicide in Korea brought attention to workplace bullying, which was pointed out as the cause. In Korea, 11.2%–23% of nurses reported experiencing workplace bullying [8]. Nurses who experience bullying have adverse effects on their physical health, such as insomnia, headache, abdominal pain, back pain, and chronic fatigue. Psychologically, bullying cause low self-confidence, anxiety, and depression, and in severe cases, it can lead to suicide. According to a meta-analysis on interventions for workplace bullying [9], individual interventions can improve bullying awareness and skill and knowledge of bullying management, which can, in turn, decrease turnover intention; Multilevel interventions were also found to be effective in eliminating bullying, as the team approach increased nurse interactions and group cohesion [9].

## Directions for improvement

### Breaking a vicious cycle

In 2015, the number of practicing nurses per 1,000 population in Korea was 5.9, only 31% of licensed nurses, contrasting with the OECD average was 9.1, which is 69.5% of licensed nurses [6]. This is due to the immediate resignation of new nurses and the in non-metropolitan areas in Korea. Although the supply of new nurses continued to increase, the number of new nurses who could not adapt to their work has also increased and led to a vicious cycle of continuous supply and leakage, comparable to continuing a blood transfusion without eliminating the cause of the bleeding. There is a fundamental problem that the immediate resignation of new nurses leads to a shortage of nurses in quantity, and at the same time, a shortage of experienced nurses, which lowers the overall quality of nursing. While accommodating the lessons of history, the direction of nursing workforce policy should be shifted toward increasing the adaptation of new nurses. This is an effective approach to securing the quantitative and qualitative levels of nurses. In Korea, preceptors have to train new nurses while taking care of their own patients. New nurses could not be trained as needed and were left unprepared to care for their patients and many of them resigned quickly within a year, and the term ‘emergent resignation’ arose. As such, it is necessary to have nurse preceptors free from the burden of caring for patients to be able to devote themselves to teaching new nurses adapt to their jobs.

Ensuring equitable working conditions and recognition is also needed. The Korean government expanded admissions to nursing schools in non-metropolitan areas to address the shortage of nurses in small and medium-sized hospitals [10]. However, after graduation, new nurses flocked to the metropolitan area where working conditions were relatively better, and regional nurse staffing did not improve. Ultimately, the key to solving the problem is providing sufficient economic compensation to nurses in non-metropolitan hospitals, improving working conditions, and ensuring stable professional development opportunities. Now, voices are calling for a shift in employment retention policies. An approach is needed to induce hospitals to increase the nurse-patient ratio by providing incentives to hospitals that comply with the Medical Act on Nursing Personnel, and significantly differentiating the hospitalization fee according to the level of nursing personnel in the health insurance system.

### Formal and informal support systems

The leadership of the head nurse is crucial in forming a mutually supportive organizational culture. However, a prior study [3] noted that nurses perceived that the head nurses were more interested in the nurses’ work and not in the nurses’ well-being. Although interested in nurses’ health, head nurses were unable to improve the situation due to too few staff, work pressure, and lack of support from hospital administrators [3]. The head nurses must take a supportive attitude toward nurses’ balance, as they are in a position to use their influence to this end. While there may be instances of avoiding acting as advocates because they do not want conflict with doctors or hospital administrators, as head nurses, they must challenge their views of appropriate actions and prepare to confront doctors and hospital administrators for the necessary changes.

Despite physical and emotional exhaustion, Korean nurses may be reluctant to admit that they need support, fearing evaluation of weakness. They lack the opportunity to heal by talking about their experiences. Therefore, it would be helpful to provide a social and emotional support system where nurses can discuss social and emotional problems in their work through regular meetings. In particular, when new nurses find it challenging to adapt to work and their self-esteem is low, meeting with fellow nurses will be an opportunity to increase their resilience while confirming that they are not alone in their experiences. These meetings will facilitate nurses to protect each other, as they can find coworkers in crisis, ask if they are well, and encourage them to use resources to help solve problems. Educational programs for nurses experiencing burnout, depression, and bullying are also needed, as well as early screening and intervention. Web-based programs will be advantageous in protecting personal information.

In Korea, a rigid recruitment system in which hospitals recruit nurses annually at a similar time is in operation. Hospitals cannot respond flexibly to nurse vacancies, and nurses have no choice but to sacrifice personal life. Vacancies in nurses who have to be absent from work due to pregnancy, illness, school, or family reasons, have no choice but to replace their colleagues. It is also worth considering establishing nurse agencies that can connect hospitals seeking to hire nurses and nurses who need jobs.

### Self-care as an ethical obligation

When nurses are socialized to accept inequality because they are women, nurses can confuse the nursing of caring with sacrifice in their relationship with the client. As if reflecting this, most of the Code of Ethics for Nurses in Korea consist of obligations to clients.

Gilligan’s seminal work [11] in women’s moral development presents that the final stage is mutual respect for self and others. In other words, recognizing that human relationships are reciprocal, women resolve the conflict between selfishness and responsibility through a new understanding of the connection between self and others. As female care workers, nurses are demanded by society to sacrifice and embody altruism. However, this cannot be done at the detriment of nurses’ lives and development. For a harmonious life, nurses need to pay more attention to their mental, physical, emotional, and spiritual health. The American Nurses Association Code of Ethics for Nurses [12] states that nurses must accept self-care as a duty to self in addition to their obligation to care for patients. Watson’s theory of human caring also provides support for nurses’ engagement in self-care [13].

Health right is a fundamental human right that everyone should enjoy, and nurses are no exception. Korean society should pay more attention to nurses’ dis-eases and health issues, recognizing them as a high-risk occupational group. Researchers in women’s health nursing should carefully study nurses’ health, so that the accumulated knowledge will guide policy and establish tangible support for nurses’ health.
